# Case Report: Limb-threatening desmoid-type fibromatosis of the foot requiring transtibial amputation

**DOI:** 10.3389/fsurg.2026.1950117

**Published:** 2026-10-29

**Authors:** Pierre-Antoine Debordes, Ceyran Hamoudi, Bruno Mota, Matteo Fortunato, Viola Sbampato, Andreas Tsoupras, Noelle Weingertner, David Brinkert, Dimitri Ceroni

**Affiliations:** 1 Pediatric Orthopedics Unit, Pediatric Surgery Service, University Hospitals of Geneva, Geneva, Switzerland; 2 Department of Orthopedics and Traumatology Surgery, Fribourg Cantonal Hospital, Fribourg, Switzerland; 3 Department of Pathology, University Hospital of Strasbourg, Strasbourg, France

**Keywords:** *CTNNB1* mutation, desmoid fibromatosis, desmoid tumor, foot tumor, lower-limb amputation

## Abstract

Desmoid-type fibromatosis (DTF) is a rare myofibroblastic/fibroblastic neoplasm characterized by infiltrative growth and a tendency towards local recurrence. In its extra-abdominal form, DTF arises in the extremities, head, neck and trunk, and the chest wall, but Desmoid fibromatosis in the foot is exceptionally rare. We present the case of a 41-year-old man who presented with recurrence of a right foot tumor initially diagnosed as a benign spindle cell lesion. The diagnosis was revised to desmoid-type fibromatosis based on a CTNNB1 p.T41A mutation. Despite systemic therapies, the tumor progressed locally, requiring transtibial amputation. Foot involvement in DTF is uncommon and challenging due to anatomical constraints. Even though this condition is not considered to be malignant, the natural history of DTF may be totally unpredictable warranting radical surgical management. This case emphasizes the importance of early diagnosis and multidisciplinary management, as delayed care may result in limb-threatening progression requiring salvage amputation.

## Introduction

Desmoid-type fibromatosis (DTF), also referred to as desmoid tumor or aggressive fibromatosis, is a rare monoclonal fibroblastic/myofibroblastic neoplasm characterized by infiltrative local growth and an unpredictable clinical course. Although DTF lacks metastatic potential, its locally aggressive behavior may result in progressive invasion of adjacent muscles, tendons, nerves, vessels, or visceral structures, leading to substantial functional morbidity. The World Health Organization classifies DTF as an intermediate, locally aggressive fibroblastic/myofibroblastic neoplasm. Its estimated incidence is approximately 3–5 cases per million person-years, with a peak incidence between 30 and 40 years of age and a slight female predominance. DTF may occur in the abdominal wall, intra-abdominal tissues, or extra-abdominal soft tissues, including the chest wall, head and neck, shoulder girdle, and extremities. Distal extremity involvement, and particularly localization in the foot, is exceedingly uncommon and may represent a major therapeutic challenge because of the limited soft-tissue envelope and the close proximity of the tumor to functionally critical anatomical structures (1–3).

The pathogenesis of DTF is primarily driven by dysregulation of the Wnt/*β*-catenin signaling pathway. Approximately 85%–90% of sporadic tumors harbor somatic activating mutations in *CTNNB1*, which encodes *β*-catenin, whereas a smaller proportion are associated with germline *APC* mutations, particularly in the setting of familial adenomatous polyposis. *CTNNB1* mutations most commonly affect exon 3, particularly codons 41 and 45. These mutations impair *β*-catenin phosphorylation and degradation, resulting in its stabilization and nuclear accumulation and subsequent transcriptional activation of genes involved in fibroblast proliferation and tumor progression. Nuclear *β*-catenin expression and identification of a characteristic *CTNNB1* mutation can therefore be particularly useful in confirming the diagnosis in challenging spindle-cell lesions. The p.T41A mutation identified in our patient is one of the most frequently reported *CTNNB1* alterations in sporadic DTF (4, 5).

The clinical course of DTF is highly heterogeneous, ranging from spontaneous stabilization or regression to progressive and locally destructive disease. This unpredictable natural history has fundamentally changed the therapeutic approach over the past decade. Active surveillance is now generally considered the preferred initial strategy for asymptomatic or minimally symptomatic patients, whereas active treatment is reserved for progressive, symptomatic, function-threatening, or anatomically critical disease. When treatment is required, management should be individualized within a multidisciplinary sarcoma team and may include systemic therapies such as tyrosine kinase inhibitors, low-dose chemotherapy, or *γ*-secretase inhibitors, as well as selected local treatments including surgery, cryoablation, or radiotherapy. Surgery is no longer routinely recommended as first-line treatment because achieving adequate margins may result in substantial functional morbidity, particularly in anatomically constrained locations (1, 3, 4).

We report the case of a 41-year-old man with recurrent extra-abdominal DTF of the foot, initially diagnosed as a benign spindle-cell lesion, which subsequently progressed despite several therapeutic approaches and ultimately required transtibial amputation. This case illustrates the diagnostic difficulties and therapeutic challenges associated with DTF arising in the foot and highlights the potential consequences of delayed diagnosis and progressive local disease.

### Case report

A 41-year-old patient from Morocco, without access to continuous healthcare coverage, presented with a two-year history of a recurring tumor of the right foot. Anamnestically, the patient reported a history of trauma to the right foot before the appearance of the tumor. A biopsy was performed, and the lesion was initially diagnosed as a “spindle cell tumor without malignancy”. A resection was performed in Morocco in 2016, but, according to the patient, local recurrence occurred rapidly after first surgery. Physical examination revealed a painless mass on the dorsum of the foot, centered on the distal third of the previous surgical scar, with a 4 cm induration. The patient experienced significant difficulty putting on his shoes. Faced with the therapeutic impasse confronting him, he came to France for a second opinion. A percutaneous biopsy under local anesthesia was performed during consultation to rule out a suspected low-grade sarcoma, due to the initial diagnosis considered and the clinical recurrence. Immunochemistry showed focal nuclear positivity for beta-catenin (clone 14) but negativity for ps100 (clone EP32) and desmin (clone NCL-DE-R-11) (Figure 1). Molecular analysis identified a CTNNB1 exon 3 p.T41A mutation (Figure 1). These findings, combined with clinical presentation, were consistent with a diagnosis of desmoid tumor.

**Figure 1:**
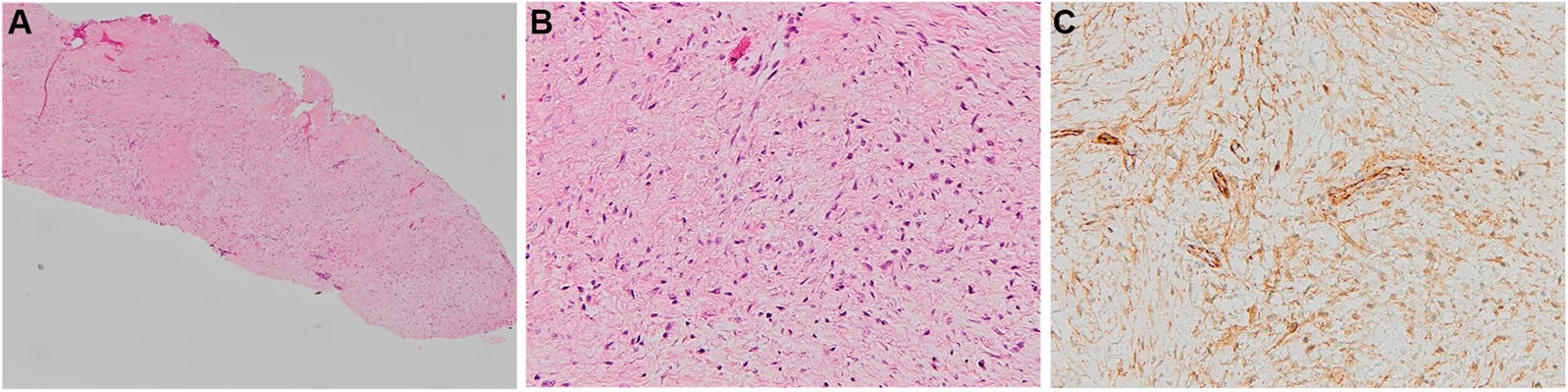
**(A)** Cells Expression for HEX 2, **(B)** Cells Expression for HEX 10, **(C)** Histopathology - T41A mutation in the beta-catenin gene exon.

An experimental first-line treatment with erythromycin (100 mg twice daily for 3 months) was initiated. The rationale for this treatment was based on the previously reported potential antiproliferative and anti-fibrotic effects of macrolides in fibroblastic proliferative disorders, including desmoid-type fibromatosis. However, no clinical improvement was observed after 3 months of treatment, and erythromycin was subsequently discontinued.

Given the aggressive local recurrence, it was decided in a multidisciplinary meeting not to propose a new surgical resection as a first-line treatment. Instead, chemotherapy was suggested, but the patient declined due to its unaffordable cost. The patient returned to Morocco for alternative treatment and was subsequently lost to follow-up. One year later, he presented to the emergency department with an ulcerated tumor augmented in size (Figure 2). MRI showed a tumor with low signal in T1 images, and high signal in T2 images, centered on the second and third metatarsals. On the plantar side, the tumor was in contact with the flexor tendons of the toes and common plantar digital nerves. On the dorsal side, the tumor extended up to the subcutaneous tissue (Figure 3). Treatment with metronomic oral vinorelbine NAVELBINE®, 30 milligrams three times per week, was started for 3 months. Initial response was favorable, leading to continue the treatment for 1 year in total. However, subsequent progression required additional strategies, including cryotherapy as part of a study (CRYODESM001). An MRI performed in Morocco showed marked tumor progression, now measuring 257 × 93  × 70 mm (Supplementary Video).

**Figure 2:**
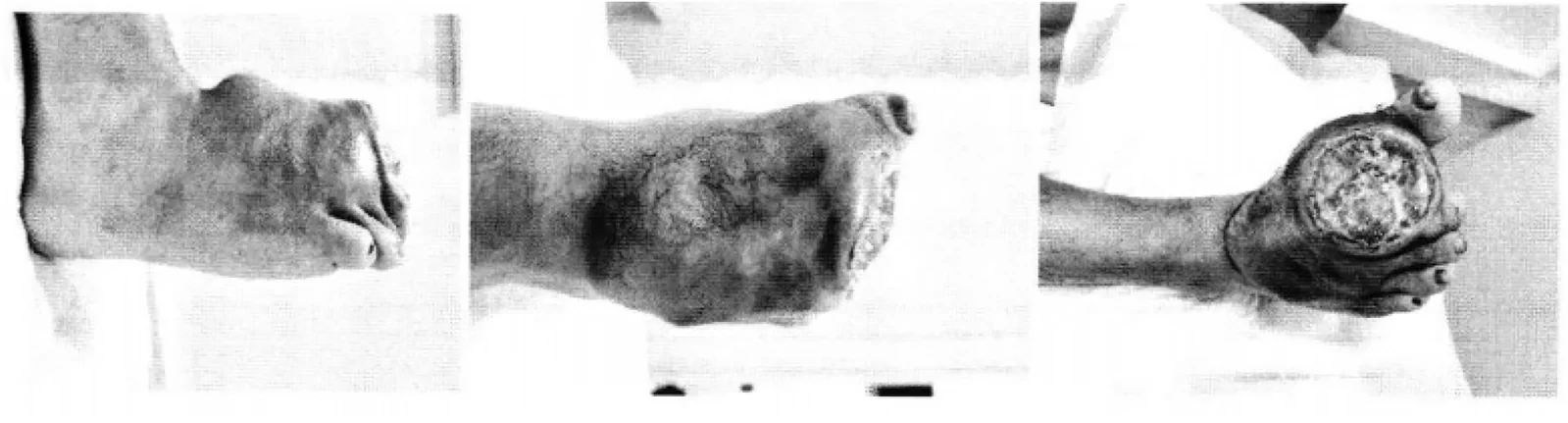
Lesion of the foot 4 years after first resection and before chemotherapy.

**Figure 3:**
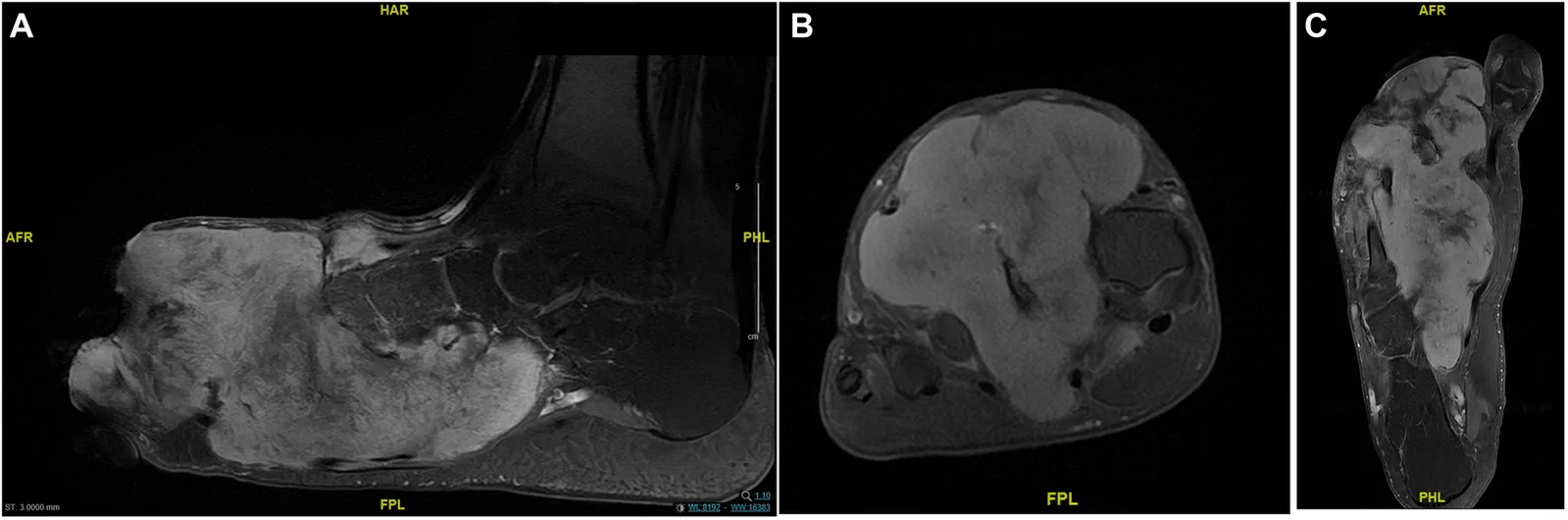
**(A)** fat-saturated post gadolinium T1 MRI of the foot – sagittal view, **(B)** fat-saturated post gadolinium T1 MRI of the foot – axial view, **(C)** fat-saturated post gadolinium T1 MRI of the foot – coronal view.

In view of the repeated treatment failure and the unfavorable disease evolution, a transtibial amputation was proposed in 2019. The patient postponed the procedure several times. The decision to proceed with transtibial amputation was ultimately made in the context of extensive local disease, with MRI demonstrating close involvement of critical anatomical structures, thereby precluding a realistic prospect of durable limb-sparing treatment. This decision also took into consideration the limited clinical benefit and suboptimal adherence associated with previous therapeutic strategies, as well as the patient's expressed preference for a definitive treatment aimed at achieving prompt and sustained disease control. The substantial financial burden of ongoing treatment, which had to be borne entirely by the patient, was an additional consideration. Within this complex clinical and socioeconomic context, transtibial amputation was deemed the most appropriate and sustainable option, offering definitive local control while providing the patient with the best prospect of timely rehabilitation and return to his professional activity. After further local worsening of the tumor, the procedure was performed a year and a half after the initial proposal (Figure 4). The amputation stump healed after an initial period of skin suffering and blistering (Supplementary Figure S1). At the six-month follow-up, there was no evidence of local recurrence. The patient reported no phantom limb pain. At 6 months from the amputation, the patient was able to resume his work as a grocer.

**Figure 4:**
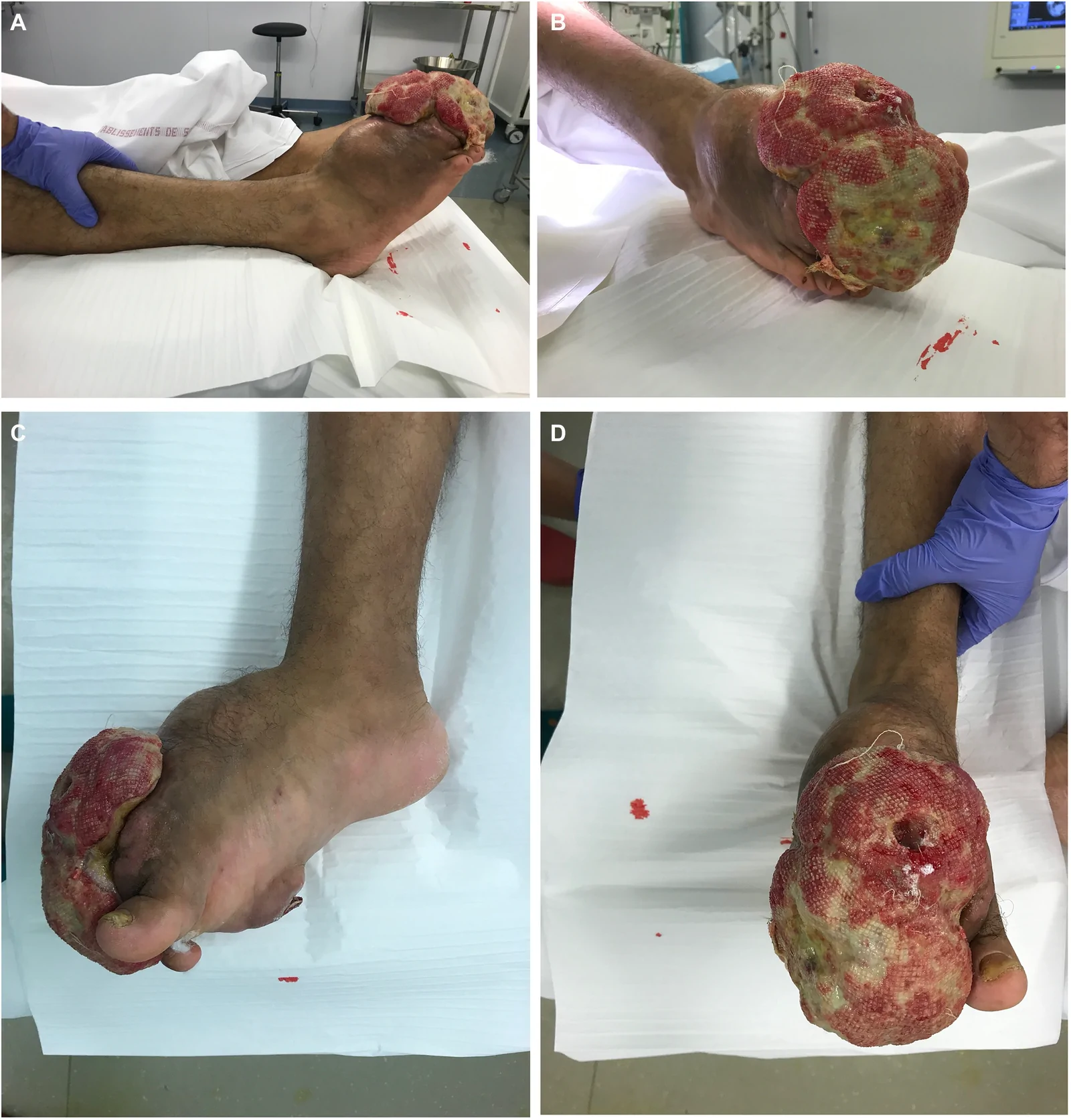
**(A)** Lesion of the foot with a global view before surgery, **(B)** Lesion of the foot with lateral view before surgery, **(C)** Lesion of the foot with medial sagittal view before surgery, **(D)** Lesion of the foot with frontal view before surgery.

## Discussion

Desmoid-type fibromatosis occupies a unique position among soft-tissue tumors. Although histologically benign and characterized by the absence of metastatic potential, these tumors may display highly aggressive local behavior, leading to significant morbidity through progressive invasion of adjacent structures. The World Health Organization classifies desmoid tumors as intermediate, locally aggressive fibroblastic neoplasms, reflecting their capacity for local destruction despite the absence of metastatic spread (5, 6).

Desmoid tumors are rare, with an estimated incidence of approximately 5–6 cases per million individuals per year, and their clinical course is highly variable. They most commonly arise in the abdominal wall, mesentery, and proximal extremities, whereas distal extremity involvement, particularly of the foot, remains uncommon (7, 8). The foot represents a particularly challenging anatomical location because of the limited soft-tissue envelope, weight-bearing function, and close relationship between tumors and critical structures such as tendons, nerves, and vessels. In advanced cases, local progression may result in severe functional impairment despite the absence of metastatic disease (9).

Accurate diagnosis requires a combination of clinical evaluation, imaging, histopathological examination, and molecular analysis. Magnetic resonance imaging plays a central role in evaluating tumor extension, anatomical relationships, treatment planning, and surveillance (6). In our patient, MRI demonstrated extensive progression involving the forefoot, with contact with flexor tendons and plantar digital nerves, limiting the possibility of functional limb-sparing treatment. Histological diagnosis may be challenging because desmoid tumors can mimic other spindle-cell lesions, including low-grade sarcomas. Diagnosis relies on the identification of a bland fibroblastic proliferation with abundant collagenous stroma, low mitotic activity, and characteristic molecular alterations. Nuclear *β*-catenin expression and CTNNB1 exon 3 mutations, particularly p.T41A, are frequently observed in sporadic desmoid tumors and strongly support the diagnosis (10, 11). In our patient, focal nuclear *β*-catenin positivity and identification of a CTNNB1 p.T41A mutation supported the diagnosis of desmoid-type fibromatosis.

The management of desmoid tumors has substantially evolved over the past decade. Historically, surgery with wide margins was considered the standard treatment because of the locally aggressive nature of these lesions. However, recognition of their unpredictable natural history, including spontaneous stabilization or regression in a significant proportion of patients, has shifted management toward individualized multidisciplinary management (6–9). Current international consensus recommendations support active surveillance as the preferred initial approach for most asymptomatic or minimally symptomatic patients, with regular clinical and magnetic resonance imaging follow-up (6, 9). Active treatment is generally reserved for progressive, symptomatic, function-threatening, or anatomically critical lesions.

When treatment is required, systemic therapy has become an important component of first-line treatment. Current therapeutic options include tyrosine kinase inhibitors such as sorafenib and pazopanib, low-dose chemotherapy regimens combining methotrexate with vinblastine or vinorelbine, and more recently *γ*-secretase inhibitors such as nirogacestat. These treatments have demonstrated improvements in progression-free survival, symptom control, and quality of life in patients with progressive or refractory disease (9, 12–16). Radiotherapy may also be considered in selected unresectable or recurrent cases when surgery would result in unacceptable morbidity or when systemic therapies are ineffective or contraindicated (17, 18). Radiotherapy can induce radiation dermatitis that is difficult to treat, especially at sites subject to high mechanical stress.

Surgery is no longer considered the routine first-line treatment but remains an option in selected patients when durable local control can be achieved without excessive functional impairment. In the foot, this balance is particularly difficult because obtaining adequate margins may require extensive resection and may result in a non-functional extremity. Therefore, surgical decisions should be individualized within a multidisciplinary sarcoma team, considering both oncological control and functional outcome (6, 9, 17).

In the present case, active treatment was justified by progressive tumor enlargement, functional impairment, skin ulceration, and failure of several therapeutic approaches. The unfavorable outcome was likely influenced by delayed diagnosis after initial excision of a lesion interpreted as a benign spindle-cell tumor, as well as limited access to specialized multidisciplinary care. The absence of healthcare coverage and financial barriers to systemic treatment contributed to interrupted follow-up and delayed treatment escalation.

Amputation for desmoid-type fibromatosis remains an exceptional procedure and should not be considered the treatment of choice for recurrence. In this case, transtibial amputation was not performed because of malignant transformation but because of progressive local destruction, repeated treatment failure, and the impossibility of achieving durable tumor control while preserving a functional foot. Despite the severity of the procedure, the patient achieved a favorable functional outcome, with independent ambulation using a prosthesis and return to professional activity six months after surgery.

This case highlights several important therapeutic lessons. Recurrent spindle-cell tumors of the foot should raise suspicion for desmoid-type fibromatosis, and early diagnosis through appropriate biopsy and molecular characterization is essential (19). Early referral to specialized multidisciplinary teams is essential, linsofar as the therapeutic decision cannot be made by the surgeon alone. This case demonstrates that, despite modern management, delayed diagnosis and persistent local progression may exceptionally result in limb-threatening disease requiring amputation.

### Patient perspective

The patient reported that the diagnosis and evolution of his disease had a significant impact on his daily life and professional activities. Initially reassured by the benign histological interpretation of the lesion, he experienced distress when the tumor rapidly recurred after the first surgical procedure. The lack of continuous healthcare coverage and limited access to specialized multidisciplinary management contributed to delays in treatment and follow-up.

As the tumor progressed, the patient developed increasing functional limitations, including difficulty wearing shoes and performing daily activities. The proposal of transtibial amputation represented a major psychological challenge, and the patient initially postponed the procedure in the hope of preserving his limb through alternative therapeutic options.

Following amputation and rehabilitation, the patient reported a significant improvement in quality of life. At six months postoperatively, he had resumed ambulation with a prosthesis and returned to his professional activity as a grocer. Despite the severity of the treatment, he considered the procedure beneficial, as it allowed definitive local control of the disease and restoration of independence.

## Data Availability

The original contributions presented in the study are included in the article/Supplementary Material, further inquiries can be directed to the corresponding author.

## Supplementary material

Supplementary Video Last MRI before transtibial amputation.

Supplementary Figure S1 Early postoperative appearance of the transtibial amputation stump showing transient skin suffering and blistering during healing.
